# Rabies Surveillance Identifies Potential Risk Corridors and Enables Management Evaluation

**DOI:** 10.3390/v11111006

**Published:** 2019-10-31

**Authors:** Amy J. Davis, Kathleen M. Nelson, Jordona D. Kirby, Ryan Wallace, Xiaoyue Ma, Kim M. Pepin, Richard B. Chipman, Amy T. Gilbert

**Affiliations:** 1 National Wildlife Research Center, United States Department of Agriculture, Animal and Plant Health Inspection Service, Wildlife Services, Fort Collins, CO 80521, USA; kim.m.pepin@aphis.usda.gov (K.M.P.); Amy.T.Gilbert@aphis.usda.gov (A.T.G.); 2 National Rabies Management Program, United States Department of Agriculture, Animal and Plant Health Inspection Service, Wildlife Services, Concord, NH 03301-8548, USA; kathleen.m.nelson@usda.gov (K.M.N.); jordona.d.kirby@usda.gov (J.D.K.); richard.b.chipman@usda.gov (R.B.C.); 3 Centers for Disease Control and Prevention, Atlanta, GA 30333, USA; euk5@cdc.gov (R.W.); hjv4@cdc.gov (X.M.)

**Keywords:** dynamic occupancy, multi-method occupancy, ORV, rabies virus, raccoon, surveillance, wildlife disease, USA

## Abstract

Intensive efforts are being made to eliminate the raccoon variant of rabies virus (RABV) from the eastern United States and Canada. The United States Department of Agriculture (USDA) Wildlife Services National Rabies Management Program has implemented enhanced rabies surveillance (ERS) to improve case detection across the extent of the raccoon oral rabies vaccination (ORV) management area. We evaluated ERS and public health surveillance data from 2006 to 2017 in three northeastern USA states using a dynamic occupancy modeling approach. Our objectives were to examine potential risk corridors for RABV incursion from the U.S. into Canada, evaluate the effectiveness of ORV management strategies, and identify surveillance gaps. ORV management has resulted in a decrease in RABV cases over time within vaccination zones (from *occupancy* ($\bar{\psi }$) of 0.60 standard error (SE) = 0.03 in the spring of 2006 to $\bar{\psi }$ of 0.33 SE = 0.10 in the spring 2017). RABV cases also reduced in the enzootic area (from $\bar{\psi }$ of 0.60 SE = 0.03 in the spring of 2006 to $\bar{\psi }$ of 0.45 SE = 0.05 in the spring 2017). Although RABV occurrence was related to habitat type, greater impacts were associated with ORV and trap–vaccinate–release (TVR) campaigns, in addition to seasonal and yearly trends. Reductions in RABV occupancy were more pronounced in areas treated with Ontario Rabies Vaccine Bait (ONRAB) compared to RABORAL V-RG^®^. Our approach tracked changes in RABV occurrence across space and time, identified risk corridors for potential incursions into Canada, and highlighted surveillance gaps, while evaluating the impacts of management actions. Using this approach, we are able to provide guidance for future RABV management.

## 1. Introduction

Intensive rabies management programs are implemented in the eastern United States and Canada to minimize the spread and eventually eliminate the raccoon variant of rabies virus. The primary method to manage the rabies virus (RABV) in wild carnivore populations is the use of oral vaccination at a landscape scale to reduce the susceptible portion of the population and consequently reduce transmission. Modeling studies focused on raccoons estimate that population immunity levels of 60%–90% may be necessary to control and eliminate raccoon RABV circulation, but ultimately levels of population immunity are sensitive to variation in host density and contact across a rural-urban continuum [1,2,3]. To vaccinate wild carnivore populations, vaccine baits are distributed in target areas defined by the host epizootiology and landscape barriers where relevant, a process referred to as oral rabies vaccination (ORV). Wildlife rabies management using ORV has been employed for close to two decades in northeastern U.S., particularly along the U.S.–Canada border [4], and occurs at a landscape scale comprising a range of habitats and raccoon densities. It is well documented that higher densities of raccoons occur in suburban and urban areas compared to rural areas [5], and the intensity of ORV targeting raccoons is scaled accordingly to raccoon density index estimates [6,7,8]. These factors may impact the effectiveness of ORV programs to control and eliminate circulation of RABV occurrence in target raccoon populations.

Two metrics are primarily used to evaluate the effectiveness of raccoon rabies management strategies. Post-baiting vaccine monitoring is conducted annually at the state level by the collection and testing of raccoon serum samples within ORV-treated areas to assess the proportion of sampled animals that have developed rabies antibodies. Enhanced Rabies Surveillance (ERS) sampling is conducted to document changes in the incidence of RABV infection in target populations. Both activities are coordinated by the United States Department of Agriculture, Animal and Plant Health Inspection Service, Wildlife Services (WS), National Rabies Management Program (NRMP) in cooperation with other agencies as described in the North American Rabies Management Plan [9]. Field trials involving animal captures both pre- and post-baiting are also conducted to document changes in the population prevalence of RABV antibodies and biomarkers, e.g., [10,11,12], but these studies are labor intensive and are usually limited in duration and spatial coverage. The NRMP utilizes information from active ERS in addition to public health surveillance data to monitor RABV incidence within and in proximity to areas managed with ORV. These data provide insight into the risk of RABV transmission across space and can determine management impacts on RABV occurrence [13,14]. There is particular interest in moving the ORV management area towards the Atlantic coast to work to eliminate raccoon RABV. These surveillance data can help identify risk corridors, or areas with higher RABV occurrence, which may provide avenues for RABV to breach the ORV barrier and where additional management or surveillance effort should be focused.

Our objectives are to examine ERS and public health surveillance data across three states in northeastern U.S. to (1) determine the dynamic occurrence of raccoon RABV over time, (2) evaluate the relationship between habitat type and raccoon RABV occurrence, (3) evaluate the impacts of the duration of ORV baiting, bait density, and bait type on raccoon RABV occurrence, and (4) evaluate the relative contributions of different surveillance methods for detecting raccoon RABV.

## 2. Materials and Methods

### 2.1. Study Area

**Figure 1:**
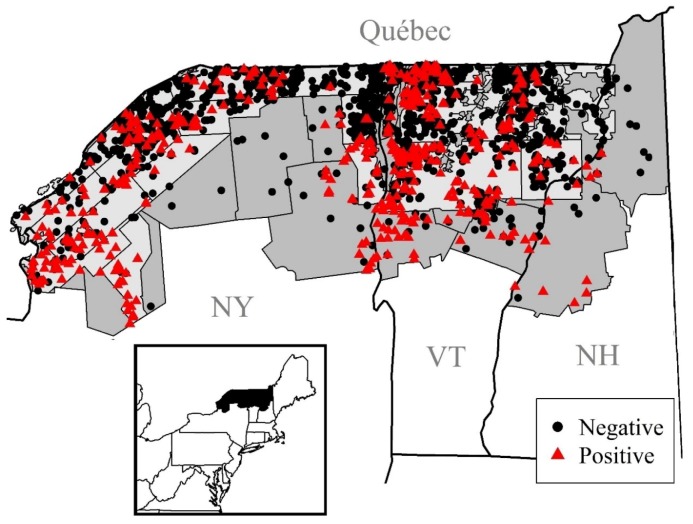
Map of the study area in northern New York, Vermont and New Hampshire. Locations of raccoons that were sampled from 2006 to 2017 are shown—the black circles were negative for raccoon variant of rabies virus (RABV) and the red triangles were positive for RABV. The oral rabies vaccination (ORV) zone for 2017 is shown as the light grey area.

Our study area encompasses northern counties in New York, Vermont, and New Hampshire that are north of 43.40 degrees latitude (Figure 1). We focused on counties that included or were adjacent to ORV areas from 2006 to 2017. There is particular interest in assessing risk corridors for new incursions of raccoon RABV into Canada [12,15]. Our study area has been intensively managed with oral rabies vaccine since 1995 and borders Quebec, Canada, making it a useful study area to examine impacts of management actions and identify risk corridors. This region (51,714 km^2^) is primarily deciduous and mixed forest (~43%), 12% evergreen forests, 6% cultivated crop cover, 13% pastureland, and 5% developed (ranging from open space to high density development) based on the 2011 National Land Cover Database [16]. The study area also includes Lake Champlain and the northern part of the Adirondack Mountains.

### 2.2. Data and Data Processing

Raccoons that were sampled for RABV as a part of ERS by the NRMP from 2006 to 2017 within the northern counties of New York, Vermont, and New Hampshire were included in our analysis. The data collected for each sampled raccoon include the location where the raccoon was sampled, date, the agency that collected the sample, how the raccoon was encountered (e.g., roadkill, surveillance trapped, and nuisance animal), and field comments. Brain tissue from each raccoon was tested for RABV using either the direct rapid immunohistochemical test (dRIT) [17] or the direct florescent antibody assay (DFA) [18]. All positive samples from WS were typed to identify the RABV variant infecting the raccoon [19]. In addition to ERS samples, we used public health surveillance data from potential human or pet exposure cases that were collected by state and local health departments, which are reported annually to the Centers for Disease Control and Prevention (CDC). State public health and veterinary laboratories all use the standard DFA test to inform recommendations for post-exposure prophylaxis [20,21].

ERS data are categorized based on the circumstances of how the raccoon was sampled. The categories are: (1) raccoons that are sick or strange acting upon sampling, (2) raccoons that were found dead by WS personnel, (3) roadkill raccoons collected by WS personnel, (4) raccoons trapped by WS specifically for RABV surveillance, (5) raccoons that were reported as nuisances by the public, and (6) sampling from any other method within ERS [22]. These classifications were formalized in the data collection process in 2016. Prior to 2016, samples were categorised post-collection based on information in the sample record, including the fate, the agency collecting the data, and comments taken during collection. Samples collected as part of public health surveillance were categorized as a separate surveillance method. We created a 10 km by 10 km grid across the study area to evaluate RABV occupancy within each grid cell. We examined data by season (calendar seasons) to accommodate both the incubation and infectious periods. Individual raccoons that were sampled by ERS and public health were either determined to be positive or negative for RABV. When a positive raccoon was sampled, the grid cell in which the raccoon was sampled (and the season it was found) was determined to be positive for RABV. Grid cells with only negative surveillance (e.g., where only negative raccoons were sampled) may have been positive for RABV and RABV was just not detected or the grid cell may have been RABV free.

### 2.3. Occupancy Analysis

Dynamic occupancy models simultaneously estimate RABV occupancy and detection [23,24]. When an area is positive for RABV, not all raccoons sampled will be positive for RABV. By comparing the proportion of samples that are positive for RABV when the area is known to be positive for RABV (i.e., at least one sample is positive for RABV across all surveillance methods), we can evaluate the probability of detecting RABV by the surveillance method using occupancy analysis [23]. When detection is not perfect (not equal to 1), we know that RABV may have been missed in some areas where it was present. By using the estimated detection probabilities, we can estimate the probability that grid cells with only negative samples were actually positive for RABV. We implemented the dynamic occupancy model using a Bayesian hierarchical model custom coded in program R [25]; for full details, see Davis et al. [26]. We used a multi-detection method approach [26,27] to estimate detection probabilities separately for each surveillance method and to account for seasonal variability in detection probabilities. Detection probabilities were estimated for each method by spring and summer separately from fall and winter to address our fourth objective. Dynamic occupancy models estimate occupancy (ψ_it_) over space “*i*” and time “*t*” by starting with estimating the initial RABV occupancy (ψ_i1_) for the first time period. Then occupancy estimate for each subsequent time period are based on the initial occupancy and whether the occupancy status at a site changed from one time period to the next (calculated by transition rates). The transition rates are colonization (γ, grid cells that are unoccupied with RABV becoming occupied in the next time step) and extinction (ε, grid cells that are occupied with RABV becoming unoccupied in the next time step). As part of dynamic occupancy, we used logistic regression to model colonization probability as a function of covariates. We examined temporal covariates (seasonal effects and a linear time trend), spatial covariates (habitat effects and elevation) and management covariates (ORV and trap–vaccinate–release (TVR) management at grid cells). The habitat effect included the percent coverage of deciduous forest cover, evergreen forest cover, cultivated crop, hay pastures, medium or high development areas, and open or low development areas based on Land Cover Database classifications from 2011 [16]. Models were validated using an area under the curve (AUC) statistic modified to accommodate the lack of detection issues as per Zipkin et al. [28].

To address our first three objectives, we used the occupancy estimates to examine the relationship between RABV occupancy probability and time, habitat composition, and management actions more explicitly in a post-hoc regression analysis. In particular, we examined how occupancy probabilities change with the duration of baiting in a given area to help provide guidance on how long baiting programs should be sustained. We used basis functions to examine non-linear relationships with bait duration [29]. Two types of bait were used in the study area during our study, RABORAL V-RG^®^ (V-RG; Boehringer Ingelheim, Athens, GA, USA), and Ontario Rabies Vaccine Bait (ONRAB; Ultralite baits, Artemis Technologies, Inc., Guelph, Ontario, Canada). There was also variability in the density of baits applied across the ORV zone ranging from 35 baits/km^2^ up to 150 baits/km^2^. In addition to ORV baiting from 2007 to 2012, localized TVR efforts were conducted within our study area. We included the log number of vaccinated raccoons from TVR efforts by grid cell and season as an additional explanatory variable for RABV occupancy. We examined the relationship between occupancy probability and habitat, seasonality, TVR management, and the ORV bait type, duration, and density using a beta regression analysis implemented using program betareg [30]. We also compared the impacts of two bait types (V-RG and ONRAB) directly using *t*-tests on the occupancy probabilities in sites prior to baiting and after one year of baiting in order to reduce complicating factors of variable bait histories. We tested whether baiting reduced occupancy for each method independently. We also compared the magnitude of occupancy reduction by bait type. These tests were conducted in program R [25].

## 3. Results

**Figure 2:**
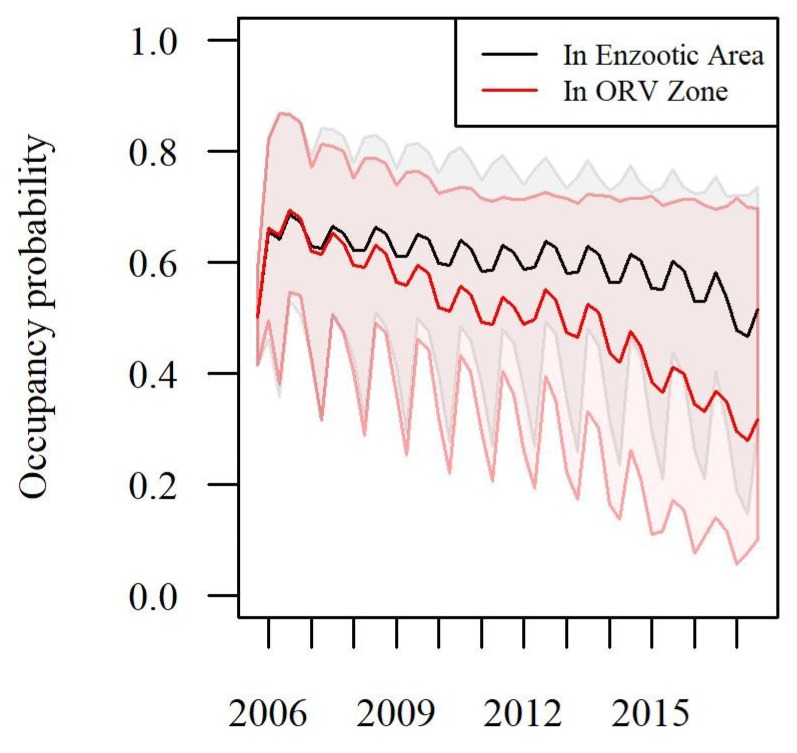
The Probability of RABV occupancy from 2006 to 2017 in the northern counties of New York, Vermont and New Hampshire. The estimates are shown by season and by status of areas within the ORV management zone (red) and those south of the ORV management zone in the enzootic area (black). The shaded regions by color show the 95% confidence intervals.

**Figure 3:**
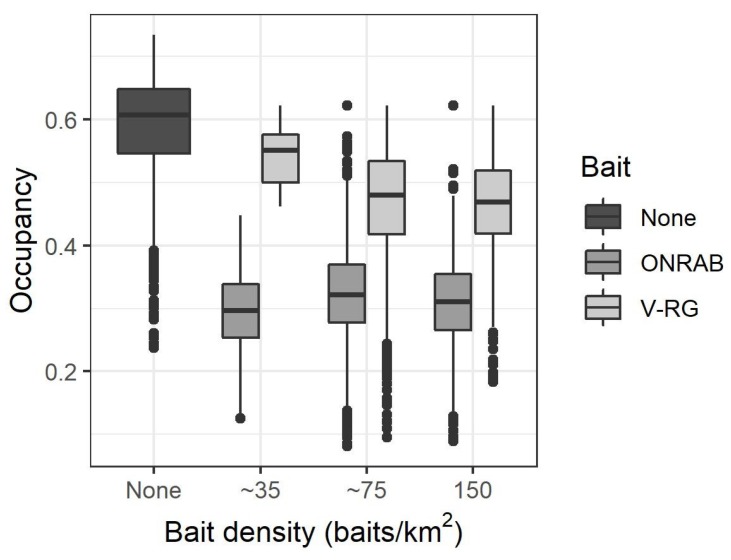
Boxplots of the probability of RABV occupancy estimates by bait density and bait type.

From 2006 to 2017, we sampled a total of 3984 raccoons in the study area. There were 503 RABV-positive samples (13% of all samples). Addressing our first objective, the occupancy analyses showed that the probability of RABV occupancy varied across seasons and generally declined over time (Figure 2). The trend across time in areas managed by ORV had lower RABV occupancy and declined more prominently than areas not managed by ORV (Figure 2, objective 3). We used *t*-tests on the posterior mean RABV occupancy estimates to compare the magnitude of occupancy reduction by bait type. The RABV occupancy in areas not managed by ORV reduced by 0.15 from the spring of 2006 to the spring of 2017 and reduced by 0.27 from the spring of 2006 to the spring of 2017 in areas managed by ORV (*p*-value < 0.001). During 1995–2011, only V-RG baits were used in the study area, whereas both V-RG and ONRAB were used from 2012 to 2017. Both V-RG and ONRAB showed significant reductions in RABV occupancy after 1 year of baiting (*p*-value < 0.001 for both bait types). ONRAB showed a greater reduction in RABV occupancy after one year of baiting compared to V-RG (a reduction of 0.12 in RABV occupancy for ONRAB compared to a reduction of 0.07 for V-RG, *p*-value = 0.001). These comparisons accounted for the fact that the average RABV occupancy prior to baiting in areas baited with ONRAB was slightly lower than for areas baited with V-RG (0.60 SE = 0.07 and 0.67 SE = 0.08 respectively). Our post-hoc beta regression analysis showed that the probability of RABV occupancy decreased as the number of years an area was baited increased (Table 1). Bait density also impacted RABV occupancy (Table 1). RABV occupancy decreased with increased bait density for V-RG but remained relatively constant for increased ONRAB bait densities (Figure 3). The TVR management strongly impacted the probability of RABV occupancy, with the occupancy decreasing with increasing numbers of raccoons vaccinated. Specifically, 50 individuals vaccinated (compared to none) related to a reduction in RABV occupancy of 0.22 (95% CI: 0.18, 0.27).

**Figure 4:**
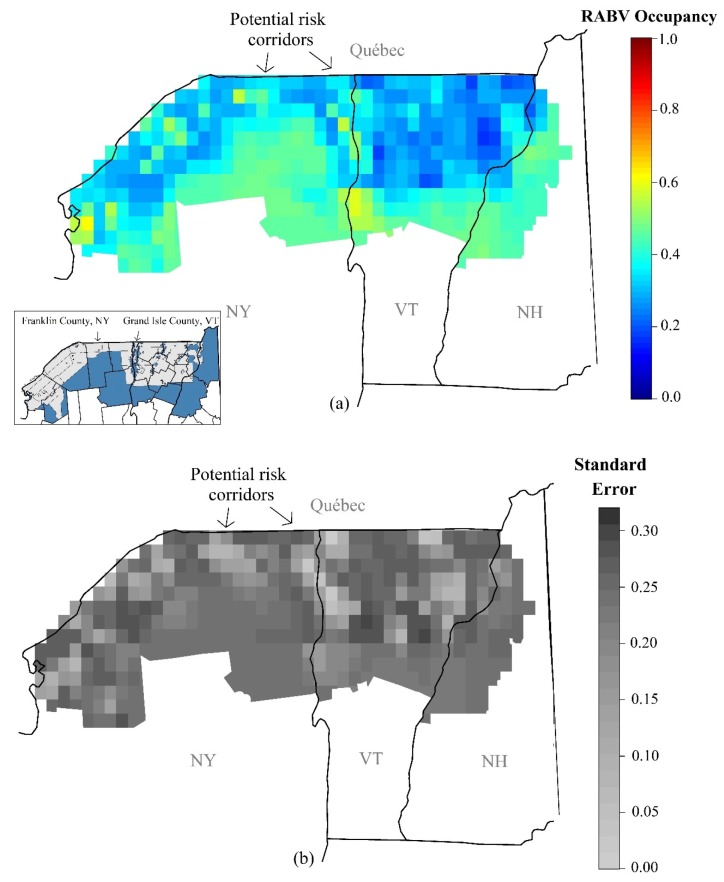
(**a**) The probability of RABV occupancy surface within the study area in the fall of 2017. The inset in this figure shows the county boundaries and the ORV boundaries for 2017 (light grey). (**b**) The standard error around RABV occupancy estimates by grid cell for the fall of 2017.

The probability of RABV occupancy varied not only by season, year and management actions, but also spatially. Habitat covariates did influence RABV occupancy, but the effects were not as strong as management actions and temporal variability (Table 1, objective 2). The strongest habitat effect was a positive relationship between the probability of RABV occupancy and the percentage of medium or high developed area (β = 1.10, SE = 0.18, Table 1). RABV occupancy declined with increased elevation (Table 1). The probability surface of the last time point can be used to identify risk corridors (Figure 4a) and identify areas with data needs (Figure 4b). Risk corridors were identified as areas with higher RABV occupancy probabilities that may provide access from the enzootic areas to Canada. Risk corridors may be areas with higher RABV probabilities or areas with lower RABV occupancy but have high uncertainty. The highest probabilities of RABV occupancy were south of the ORV management zone and tended to be associated with cities and areas of low elevation (Figure 4a). Occupancy probability in the ORV zone was lower but more variable than outside the zone and corresponded to higher uncertainty (Figure 4b).

**Figure 5:**
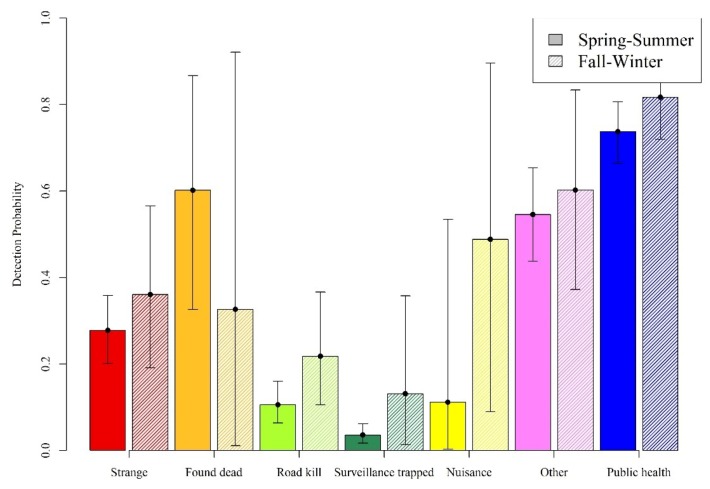
The probability of RABV detection by the surveillance method allowing for within year variability (spring to summer—solid colors; fall to winter—hashed bars). The colors correspond to surveillance method: strange acting in red, found dead in orange, roadkill in light green, surveillance trapped method in dark green, nuisance samples in yellow, other samples in violet, and public health samples in blue. The 95% credible intervals are shown per estimate.

Public health samples comprised the largest percent of all samples tested (30.75%, Table 2). Found dead and surveillance trapped were the smallest percentages of all samples (1.51% and 7.03% respectively, Table 2). The other surveillance methods ranged from 12% to 18% of all samples. Over three-quarters of the samples were collected in the spring and summer compared to the fall and winter (Table 2). Over half of all RABV-positive samples were from the public health data (59.84%). Fewer than 10% of all positive samples came from either found dead, roadkill, surveillance trapped, or nuisance collection methods. Detection probability is conditioned on RABV being present, therefore detection probabilities can be very different from raw prevalence rates. Addressing our fourth objective, we found that found dead and other samples in spring to summer, and public health and nuisance samples in fall to winter had the highest detection probabilities (Figure 5). Roadkill and surveillance trapped methods had low detection probabilities but were both higher in fall to winter compared to spring to summer (Figure 5).

We had a good model fit using the Zipkin et al. [28] AUC statistic for occupancy models (AUC = 0.97), suggesting our model adequately recaptures our estimated RABV occurrence patterns.

## 4. Discussion

One of our primary objectives was to evaluate the effectiveness of ORV as a wildlife rabies management strategy in northeastern U.S. Raccoon RABV occupancy declined (corresponding to case reduction) over time in areas within ORV management zones more substantially than in raccoon RABV enzootic areas without management. However, occupancy in the raccoon RABV enzootic areas also declined during our twelve-year study period. This may suggest a natural waning of raccoon RABV circulation in the raccoon populations of northeastern U.S. in recent years or a post-epizootic rebound effect (lower susceptible populations). It could also possibly be an added benefit of nearby intensive ORV management. Raccoons exposed to vaccination baits within management areas may migrate and intermix with populations not being actively managed. These individuals may be helping to reduce the transmission of raccoon RABV even in the absence of direct management.

Considerable effort and research have been applied to developing strategies to manage raccoon RABV. Previous studies have suggested that both the duration of baiting and the bait density influence the seroprevalence in raccoons [8,31]. Our results support the importance of these vaccination strategies. Additionally, we found support for differences in RABV occupancy based on the type of oral rabies vaccine distributed in our study area. Consistent with comparative studies based on serology [12,32,33], we found that ONRAB outperformed V-RG in reducing RABV occupancy (reducing RABV occupancy after one year of baiting in an unbaited area by 0.12 for ONRAB compared to 0.07 for V-RG). The study area was baited with V-RG exclusively for over a decade before ONRAB was applied. Furthermore, as we have determined, the baiting history in an area and the bait density are also important indicators of RABV occurrence. However, even when we look at areas with similar baiting histories and bait densities, we did find that areas treated with ONRAB had lower occupancy than areas treated with V-RG.

Serology studies on raccoons have shown variability in seroprevalence responses to baiting strategies even when using similar vaccine bait types and similar bait densities [8,34,35], suggesting that other factors may influence the effectiveness of baiting strategies. Potential complicating factors may include significant variation in raccoon densities, variation in opportunities for target species to encounter vaccine bait by habitat type, and the potential for bait competition by non-target species [36]. These variations in seroconversion response may help explain the variability in occupancy we observed. For example, we found higher RABV occupancy in areas with increased medium and high-density developed habitats. These higher occupancy rates might relate to higher densities of raccoons associated with urban areas [37,38], despite rates of bait application that are approximately doubled in urban areas compared to rural areas. Based on the results of our study, the additional optimization of baiting strategies in suburban and urban areas appears warranted.

In addition to ORV management, from 2007 to 2012 several intensive TVR management actions were conducted within our study area. TVR efforts have been shown to be effective in helping to reduce RABV cases [33,39]. Due to the intense effort associated with TVR programs, they are usually localized and conducted over short periods of time [40] and thus TVR is often used in conjunction with other approaches (e.g., population reduction or ORV) to help have a broader impact [36,40]. TVR programs in our study area were conducted primarily along the U.S.–Canada border in Vermont, along the Vermont–New York and Vermont–New Hampshire borders, and along the St. Lawrence River in New York. We found a strong impact of TVR programs on RABV occupancy, with larger numbers of raccoons vaccinated corresponding to lower RABV occupancy rates. These impacts were pronounced even though the majority of TVR activities occurred within active ORV management zones. This supports the idea that combined approaches can be more effective at reducing RABV cases [4,41].

Our study identifies areas with high probabilities of RABV occupancy which may indicate potential risk corridors for raccoon RABV transmission north into Canada. Using the spatial and temporal patterns we observed in raccoon RABV occurrence (our first two objectives), we identified two potential risk corridors, one in northern Franklin County, NY, and one on the New York–Vermont border in Grand Isle County, Vermont. These are paths from the enzootic area that are in the ORV management area and have higher RABV occupancy. The area in Franklin County, New York, had a brief outbreak of RABV in the spring of 2015, but has not been intensively sampled since then and thus remains an area of concern. The higher risk is related both to the previous history in the area and to the lack of sufficient sampling to be confident of RABV elimination. The risk corridor along the New York–Vermont border is an area of low elevation and low elevation areas generally are associated with higher raccoon densities and RABV occupancy. Raccoon densities have been reported to be lower in areas with high elevation [7,42]. If RABV transmission and spread is principally influenced by raccoon density [2,43], this may relate to the higher risk in this area.

To be able to evaluate RABV occurrence patterns, we need to understand how different surveillance methods influence our ability to detect raccoon RABV on the landscape (our fourth objective). Without accounting for the lack of detection issues, we would have biased estimates of RABV occupancy [23] and therefore may not have estimated spatial, temporal, and management relationships with occupancy adequately. Detection probabilities varied by the surveillance method, similar to previous work in Ohio, West Virginia, and Pennsylvania [26]. Public health data, which largely come from animals tested due to human, livestock, or pet exposures, had the highest detection rates in our study, as expected. Public health samples formed a disproportionately larger composition of samples from the enzootic area compared to areas being managed with ORV in our study, which may relate to the higher detection rates. Overall, we found some similar patterns in detection probabilities compared to the study in Ohio, West Virginia, and Pennsylvania (e.g., strange acting, found dead, and public health samples had higher detection rates and surveillance trapped samples had very low detection probabilities). However, this study showed higher detection in the unknown/other method of detection as well as a higher proportion of samples from this method. The classification of samples to the designated surveillance methods was not implemented in the field until 2016 [22]. The post-sampling categorization that was necessary prior to 2016 used details in the sample records (such as animal fate, collection agency, and comments) to make post-hoc assignments to the defined methods. Differences between state agency collection protocols may contribute to the higher proportion of uncategorized samples in this study compared to the study in Ohio, West Virginia, and Pennsylvania. The downside of having a larger percentage of uncategorized samples means that there is greater uncertainty in the detection probabilities where these samples should have been categorized. Detection rates across our surveillance methods in our study were higher than in our previous study during the same time period in Ohio, West Virginia, and Pennsylvania, despite only collecting approximately one-fifth of the samples relative to our previous study (3984 compared to 23,635). Variations in detection probabilities may be related to factors such as habitat, which may be useful to examine in future research.

## 5. Conclusions

Our retrospective analysis linked patterns of RABV occupancy over time to management actions at the landscape scale. We demonstrated a decrease in RABV occupancy with the duration of baiting for two bait types, and across a wide range of bait densities. However, our study design did not include data from paired vaccinated and unvaccinated sites with similar incidence and ecology, which limits our ability to infer causation. Nonetheless, our RABV data span a large spatial area that include vaccinated and unvaccinated areas. There was a much stronger rate of RABV decline in the vaccinated areas relative to the unvaccinated areas, suggesting that this result is robust. Experimental studies that measure incidence and seroprevalence pre- and post-vaccination would be a useful compliment for quantifying the magnitude of vaccination impacts and the levels of vaccination that lead to herd immunity under different ecological and RABV incidence conditions.

## Figures and Tables

**Table 1:** Covariate estimates for the beta regression model on RABV occupancy. Factors are sorted in descending order from the absolute magnitude of the effect. The duration of baiting by V-RG and Ontario Rabies Vaccine Bait (ONRAB) were modeled with basis functions (BS) to allow for non-linear effects. Their factors are grouped together and sorted based on the largest magnitude effect.

Factor	Estimate	Std. Error	z Value	Pr(>|z|)
(Intercept)	1.15	0.02	57.13	0.00
Bait density by bait type interaction	1.28	0.07	18.43	0.00
Bait density	−1.18	0.06	−20.46	0.00
Year	−0.79	0.01	−63.25	0.00
Medium to high development	0.62	0.25	2.52	0.01
ONRAB duration BS-1	−0.08	0.11	−0.73	0.46
ONRAB duration BS-2	−0.13	0.04	−3.36	0.00
ONRAB duration BS-3	−0.43	0.05	−8.00	0.00
Open to low development	−0.41	0.16	−2.61	0.01
Bait type	−0.41	0.05	−8.32	0.00
V-RG duration BS-1	0.20	0.05	3.86	0.00
V-RG duration BS-2	0.32	0.05	5.93	0.00
V-RG duration BS-3	−0.40	0.06	−6.37	0.00
Deciduous forest	−0.36	0.02	−17.05	0.02
Spring	−0.34	0.01	−45.09	0.00
Summer	−0.29	0.01	−37.89	0.00
Hay/pasture	0.26	0.04	6.61	0.00
log(# raccoons vaccinated with trap–vaccinate–release management)	−0.25	0.00	−69.24	0.00
Cultivated crops	0.15	0.07	2.33	0.02
Wetlands	0.14	0.04	3.39	0.00
Winter	−0.13	0.01	−16.53	0.00
Years of total baiting	−0.08	0.02	−3.98	0.00
Mean elevation	−0.08	0.01	−8.30	0.00
Evergreen forest	0.06	0.04	1.79	0.07

**Table 2:** Number of negative and positive RABV samples and total raccoon samples from 2006 to 2017 in northeastern U.S. by the surveillance method.

	Fall to Winter			Spring to Summer		
Method	Negative	Positive	Total	Negative	Positive	Total
Strange acting	99	12	111	388	50	438
Found dead	13	0	13	36	11	47
Roadkill	138	12	150	517	30	547
Surveillance trapped	70	1	71	608	9	617
Nuisance	25	2	27	251	2	253
Other	147	13	160	265	60	325
Public health	229	104	333	695	197	892
